# Development and Application of High-Throughput Single Cell Lipid Profiling: A Study of *SNCA-A53T* Human Dopamine Neurons

**DOI:** 10.1016/j.isci.2020.101703

**Published:** 2020-10-21

**Authors:** Stuart G. Snowden, Hugo J.R. Fernandes, Josh Kent, Stefanie Foskolou, Peri Tate, Sarah F. Field, Emmanouil Metzakopian, Albert Koulman

**Affiliations:** 1 Core Metabolomics and Lipidomics Laboratory, Metabolic Research Laboratories, Institute of Metabolic Science, University of Cambridge, Level 4 Pathology, Cambridge Biomedical Campus, Cambridge CB2 0QQ, UK; 2 Department of Biological Sciences, Royal Holloway University of London, Egham, Surrey Tw20 0EX, UK; 3 UK Dementia Research Institute, University of Cambridge, Department of Clinical Neurosciences, Cambridge Biomedical Campus, Cambridge CB2 0AH, UK

**Keywords:** Molecular Neuroscience, Cellular Neuroscience, Lipidomics, Metabolomics

## Abstract

Advances in single cell genomics and transcriptomics have shown that at tissue level there is complex cellular heterogeneity. To understand the effect of this inter-cell heterogeneity on metabolism it is essential to develop a single cell lipid profiling approach that allows the measurement of lipids in large numbers of single cells from a population. This will provide a functional readout of cell activity and membrane structure. Using liquid extraction surface analysis coupled with high-resolution mass spectrometry we have developed a high-throughput method for untargeted single cell lipid profiling. This technological advance highlighted the importance of cellular heterogeneity in the functional metabolism of individual human dopamine neurons, suggesting that A53T alpha-synuclein (*SNCA*) mutant neurons have impaired membrane function. These results demonstrate that this single cell lipid profiling platform can provide robust data that will expand the frontiers in biomedical research.

## Highlights

• Combining FACS and LESA-MS to establish high-throughput single cell lipid profiling
• Lipid differences found within and between populations of human dopamine neurons
• Inter-cell lipid heterogeneity is increased in *SNCA-A53T* dopamine neurons
• Identification and isolation of human iPSC-dopamine neurons with a TH-RFP reporter

## Graphical Abstract

**Figure:**
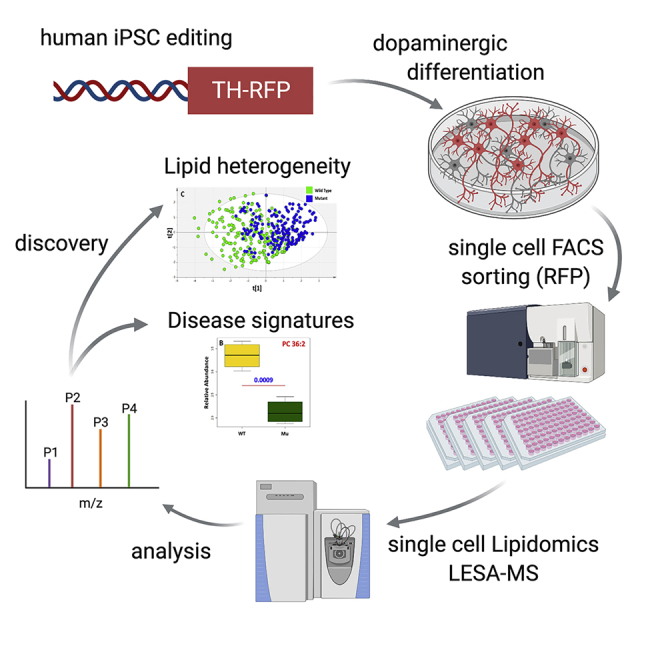

## Introduction

The recent developments of single cell approaches have demonstrated the importance of measuring and understanding how cellular heterogeneity affects tissues and organs to fully understand biological processes (Heintzman et al., 2009; Tiklova et al., 2019; Tirosh et al., 2016; Deng et al., 2014). Single cell genomic and transcriptomic approaches have enabled previously unanswered questions to be addressed. For example, Tiklova et al*.* performed single cell RNA sequencing of *pitx3* mouse midbrain dopamine neurons and identified seven distinct neuronal subtypes, five of which expressed dopaminergic markers and five expressed glutamatergic and GABAergic markers (Tiklova et al., 2019). However, to date there has been limited success in the development of practical metabolic phenotyping tools that can be applied to capture the metabolic heterogeneity at a cellular level.

Lipids are the most abundant class of metabolites in the cell, and the measurement of lipids by mass spectrometry in bulk samples is well described. A handful of studies have previously described proof of principle for single cell lipid profiling (Evers et al., 2019); however, these are not platforms capable or suitable for robust high-throughput readouts of cell activity. Ellis et al*.* used a low-throughput approach where cell droplets were printed onto a glass slide, which were imaged and analyzed using liquid extraction surface analysis coupled with mass spectrometry (LESA-MS) (Ellis et al., 2012). Neumann et al*.* used MALDI to measure lipids from a large number of putative single cells from a section of rat cerebellum. However, without imaging all samples lack cell-type specificity and could not guarantee that each sample contained a single cell and not clusters of cells, leading to wide divergence in the number of lipids measured per sample (Neumann et al., 2019). Most single cell mass spectrometry platforms have focused on analyzing immobilized cells; however, Standke et al. (2019) developed an integrated cell manipulation platform that enables single cells to be analyzed from solutions, such as bodily fluids, with minimal sample preparation. More complex derivatization approaches have also been described. Thiele et al. (2019) reported a method for tracing lipid metabolism in cell culture dilutions using click chemistry. This provided detailed coverage but was also unable to give certainty that bona fide single cells were analyzed. Together, these studies are convincing proofs of principle, but they do not represent mature platforms. To achieve this, it is necessary to standardize sample handling, single cell isolation and to establish robust strategies for quality control to ensure that the generated data can be meaningfully compared.

The brain is a lipid-rich organ, and neuronal lipid metabolism regulates a range of biological processes including cell signaling and structural integrity (Tracey et al., 2018). Perturbations of lipid metabolism have been associated with the pathogenesis of Parkinson disease (PD), from genetic risk factors to altered brain lipid profiles (Do et al., 2011; Fabelo et al., 2011). PD is a common neurodegenerative disorder characterized by the loss of dopamine neurons and the accumulation of Lewy bodies, which are composed primarily of alpha-synuclein protein (Spillantini et al., 1997). The relevance of *SNCA* for the pathology of PD is further highlighted by the fact that mutations in this gene, such as the A53T mutation, lead to the development of PD (Polymeropoulos et al., 1997). Although the underlying mechanisms of this association are unclear, alpha-synuclein has been shown to modulate lipid metabolism in PD models (Golovko et al., 2007; Sharon et al., 2003; Zambon et al., 2019).

**Figure 1:**
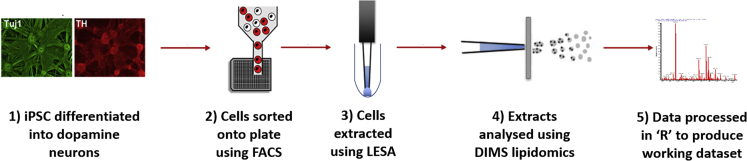
Graphical Representation of the Analytical Single Cell Pipeline Used in this Study

Here we describe the development of a high-throughput (280 single cells/day) untargeted single cell lipid profiling platform, detailing strategies for single cell isolation, data generation, automated signal identification, and quality control (Figure 1). We further demonstrate that this is a mature platform by validating the measured lipids in three independent cohorts and through its application to the analysis of human dopamine neurons derived from induced pluripotent stem cells (iPSCs). We believe this important technological advance for single cell lipid analysis will provide a unique opportunity to address important biomedical questions across various fields of research.

## Results

### Measuring Lipids in Single Cells

Protocols for the differentiation of *in vitro* human dopamine neurons are known to generate heterogeneous neuronal populations (La Manno et al., 2016). To overcome this, we engineered human wild-type (WT) iPSC with a tyrosine hydroxylase (TH) red fluorescence protein (RFP) as previously described (Xia et al., 2017), with modifications. To express tagRFP-T under the influence of the endogenous TH promoter we introduced a P2A-tagRFP-T donor plasmid with homologous arms flanking the TH stop codon and an EF1A-Puromycin selection flanked by *LoxP* sites for drug selection. The targeting plasmid was introduced into iPSCs together with a single guide RNA targeting the 3′ end of the TH gene and Cas9 RNP by nucleofection to cause a double-strand break near the stop codon. After Puromycin selection, transient expression of Cre was used to excise the Puromycin cassette. TH catalyzes the hydroxylation of L-DOPA (the rate-limiting step in the synthesis of dopamine) and is considered a bona fide marker for dopamine neurons. After differentiation into dopamine neurons (Siddiqi et al., 2019; Kriks et al., 2011), fluorescence-activated cell sorting (FACS) was used to sort cells based on RFP expression. RFP-positive dopamine neurons were individually sorted into multi-well plates, followed by LESA-MS.

**Figure 2:**
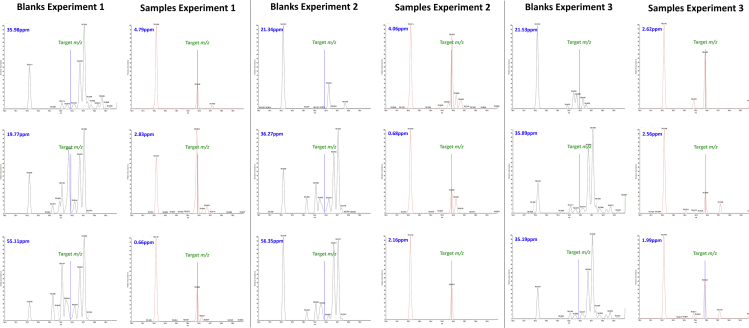
Spectra Showing the Presence of a Signal for PC 34:1 in Single Cell Samples and Its Absence from Extraction Blanks in Three Independent Experiments Each spectra shows the location of the “target *m/z*” and the deviation from the “target m/z” of the closest measured signal.

In spectra generated from single cell samples (Figure 2) there was a clear signal for PC 34:1 with an average deviation of 2.6 ppm and with the highest deviation being 4.8 ppm. However, in the blanks there was no signal for PC 34:1 with a mean deviation between the closest signal and the target mass of 30.8 ppm and the lowest deviation of 15.1 ppm. Comparison of the relative abundance and deviation of the single cell samples to the blanks showed that the samples have a significantly higher abundance and lower deviation (Table S1). Having identified PC 34:1 and other lipids in initial analyses we performed two additional independent experiments to validate these findings. Across these experiments the average deviation in the blanks was 17.8 ppm with only 4 of 63 blanks having a signal falling within 10 ppm of the target mass, whereas in single cells the average deviation was 1.8 ppm and the highest deviation was 7.8 ppm with signals detected in over 90% samples.

Next, we determined how many lipids could be detected in the three datasets. In each dataset we applied four QC filters, signal falling within 10 ppm of the expected mass, a signal-to-noise ratio greater than 1.5, a linear relationship between lipid signal and cell number (r > 0.5) in a cell “standard” curve, and a signal had to be present in at least 5% of single cell samples. Once we had identified the lipids measured in each of the three datasets we defined three levels of confidence in the putative assignments. Level 1 putative assignments are lipids that were successfully measured in all three datasets, level 2 putative assignments were present in two of the three, and level 3 assigned lipids were only measured in a single experiment. We successfully identified 37 level 1 lipid assignments corresponding to 25 glycerophosphocholines (PCs), 9 glycerophosphoethanolamines (PE), and 3 sphingomyelins (SM) (Table 1). A further 23 level 2 and 21 level 3 putative assignments were also identified including an additional 15 PCs, 22 PEs, and 1 SM as well as 2 ceramides, 1 diglyceride, 3 glycerophosphoserines, and 1 triglyceride (Table S2)

**Table 1:** Panel of Lipids Successfully Measured from a Single Dopaminergic Neuron

	Experiment 1	Experiment 2	Experiment 3
PC 30:1	33.3	57.5	24.3
PC 32:0	66.7	65.8	71.6
PC 32:1	88.9	65.0	67.8
PC 32:2	11.1	65.0	45.9
PC 32:3	66.7	55.0	30.0
PC 32:4	44.4	46.3	23.9
PC 34:0	66.7	15.0	30.2
PC 34:1	100.0	94.9	90.2
PC 34:2	88.9	86.1	60.8
PC 34:3	66.7	88.8	68.5
PC 34:4	77.8	93.7	78.2
PC 36:1	77.8	89.9	74.7
PC 36:2	88.9	88.6	88.4
PC 36:3	66.7	82.2	65.3
PC 36:4	88.9	91.3	86.4
PC 36:5	66.7	78.7	37.2
PC 38:3	88.9	78.8	39.2
PC 38:4	55.6	67.6	44.4
PC 38:5	88.9	77.5	65.5
PC 38:8	44.4	21.3	6.3
PC 40:3	44.4	58.1	36.9
PC 40:4	22.2	33.8	27.7
PC 40:5	22.2	17.5	42.6
PC 40:6	11.1	7.5	58.1
PC 40:9	11.1	5.0	30.0
PE 34:0	28.6	15.0	7.7
PE 34:1	44.4	46.8	30.6
PE 34:2	22.2	16.5	13.3
PE 34:3	11.1	13.9	11.3
PE 36:0	22.2	17.5	8.6
PE 36:1	44.4	32.9	30.9
PE 36:3	11.1	11.4	25.7
PE 36:4	11.1	10.1	7.9
PE 38:6	22.2	15.0	18.6
SM 34:1	33.3	37.7	30.6
SM 36:1	22.2	29.5	18.9
SM 36:2	22.2	37.5	7.9

Data showing the percentage of cells in which each lipid was measured in each of the three sample sets. PC, glycerophosphocholine; PE, glycerophophoethanolamine; SM, sphingomyelin.

### Quality Control

**Figure 3:**
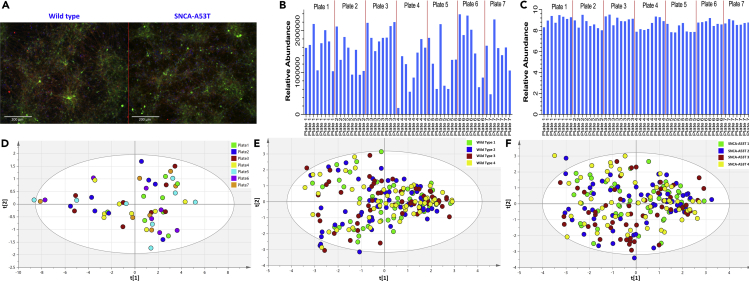
Plots Showing the Lipid Abundance in Quality Control Samples from all Seven Plates Both Before and After Batch Correction (A) Immunofluorescence staining of WT iPSC-differentiated dopamine neurons showing expression of pan-neuronal marker beta-3-tubulin (TUJ1) in red, dopamine marker tyrosine hydroxylase (TH) in green, and nuclear DAPI in blue. (B) Plot of uncorrected abundance of PC 34:1 in individual QC samples. (C) Plot of the corrected abundance of PC 34:1, in individual QC samples. (D) PCA scores plot of quality control samples calculated using all corrected lipid data. Mu, *SNCA-A53T* mutant dopaminergic neurons; PC, glycerophosphocholine; PCA, principal-component analysis. (E) PCA scores plot showing the comparability of lipid composition between wild-type cell populations. (F) PCA scores plot showing the comparability of lipid composition between *SNCA-A53T* cell populations.

Having validated the platform, we expanded our method to address a relevant biological question by assessing the impact of the *SNCA-A53T* mutation in the lipidome of human dopamine neurons at the single cell level resolution. To reduce inherent variability associated with iPSC modeling studies as previously reported (Volpato and Webber, 2020), we generated isogenic *SNCA-A53T* mutant iPSC lines using CRISPR-Cas9 on a WT background as previously described (Siddiqi et al., 2019, Bruntraeger et al., 2019). Isogenic *SNCA-A53T* mutant iPSCs were then engineered with a TH-RFP reporter following the same procedure described for WT cells. Differentiation efficiency into dopaminergic neurons was similar for WT and *SNCA-A53T* iPSCs (Figure 3A). After differentiation, WT and *SNCA-A53T* individual dopamine neurons were sorted by FACS across 7 plates of 96 wells. For each genotype, a total of 280 single neurons across four biological replicates were sorted with extraction blanks and quality controls added across all plates.

When comparing the abundance of PC 34:1 measured in the QC samples from all 7 plates (Figure 3B) there was a significant (p < 0.05) difference in the abundance of this lipid between plates, with the greatest difference seen between plates 3 and 4. However, after the data were normalized to total signal abundance this difference was corrected for with no significant differences (p < 0.05) found in the abundance of PC 34:1 between plates (Figure 3C). Principal-component analysis (PCA) of the normalized lipid profiles from QC samples revealed that samples could not be clustered according to different plates, confirming a robust and standardized sample handling and processing (Figure 3D). Analysis of normalized lipid profile data using PCA showed that there was no relationship between injection order and lipid composition (Figure S1).

### Comparing Single Cell and Population Lipid Profiling

**Figure 4:**
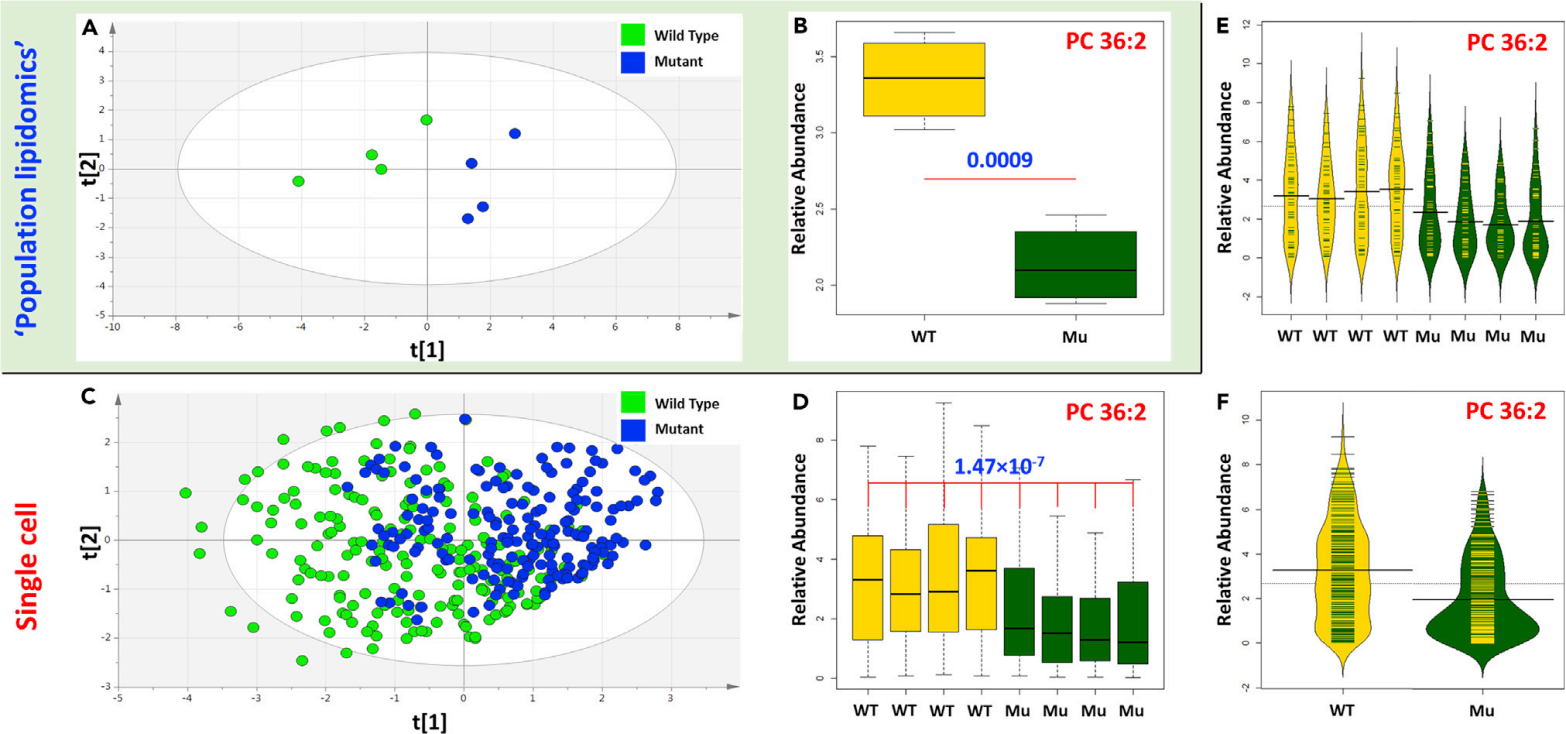
Comparison of the Information Provided by Population and Single Cell Lipid Profiling Approaches (A) PLS-DA scores plot of population level lipid profiling comparing wild-type and A53T mutants (Mu). (B) Boxplot of the abundance of PC 36:2 in wild-type and A53T mutants (Mu); p value calculated using generalized linear model. (C) PLS-DA scores plot of single cell lipid profiling comparing wild-type and A53T mutants (Mu). (D) Boxplots of the abundance of PC 36:2 in each biological replicate, p value calculated using generalized linear model. (E) Beanplot showing the relative distribution of the abundance of PC 36:2 in each biological replicate cells where each line represents the abundance measured in a cell and the width of the outline represents the density of samples. (F) Beanplot showing the relative distribution of the abundance of PC 36:2 in combining all wild-type and all mutants where each line represents the abundance measured in a cell and the width of the outline represents the density of samples. All p values were calculated using generalized linear models. Mu, *SNCA-A53T* mutant dopaminergic neurons; PC, glycerophosphocholine; PLS-DA, partial least squares-discriminant analysis.

Next, we explored the advantages of using single cell lipid profiling over traditional population level approaches in this dopamine neurons dataset. After data processing and normalization, the mean abundance of each lipid was calculated within each biological replicate to obtain a “population” lipid profile. When these “population” profiles were analyzed with partial least squares-discriminant analysis (PLS-DA) (R^2^X = 0.679 R^2^Y = 0.752 Q^2^ = 0.530 CV-ANOVA = 4.50 × 10^−6^) there was a clear difference between WT and *SNCA-A53T* populations (Figure 4A). PC 36:2 was the most important lipid driving the PLS-DA model with univariate analysis also identifying a significant difference between genotypes (p = 0.0009) (Figure 4B), with PC 36:1 and PC 34:2 also significantly different between genotypes (Figures S2 and S3). This “population” analysis implied significant lipid alterations associated with the A53T mutation and suggested non-overlapping signatures between WT and *SNCA-A53T* dopamine neurons.

However, when the lipid profiles of individual dopamine neurons were analyzed we found a considerable heterogeneity within both genotypes (Figure 4C). Although the PLS-DA scores plot (R^2^X = 0.306 R^2^Y = 0.226 Q^2^ = 0.195 CV-ANOVA = 7.65 × 10^−18^) still confirmed differences between WT and *SNCA-A53T* dopamine neurons, there was some overlap between genotypes. This cellular heterogeneity could be of fundamental importance for a complete understanding of biological function and could only be identified by single cell lipid profiling.

Looking at individual lipids the abundance of PC 36:2 was still significantly different (p = 1.4 × 10^−7^) between genotypes (Figure 4D), with differences also observed in PC 36:1, PC 34:2, and PC 32:0 (Figures S2–S4). However, there was a high degree of cellular heterogeneity (Figures 4E and 4F) with the A53T mutants showing a high density of neurons with low abundances of PC 36:2 (Figures 4E and 4F).

**Figure 5:**
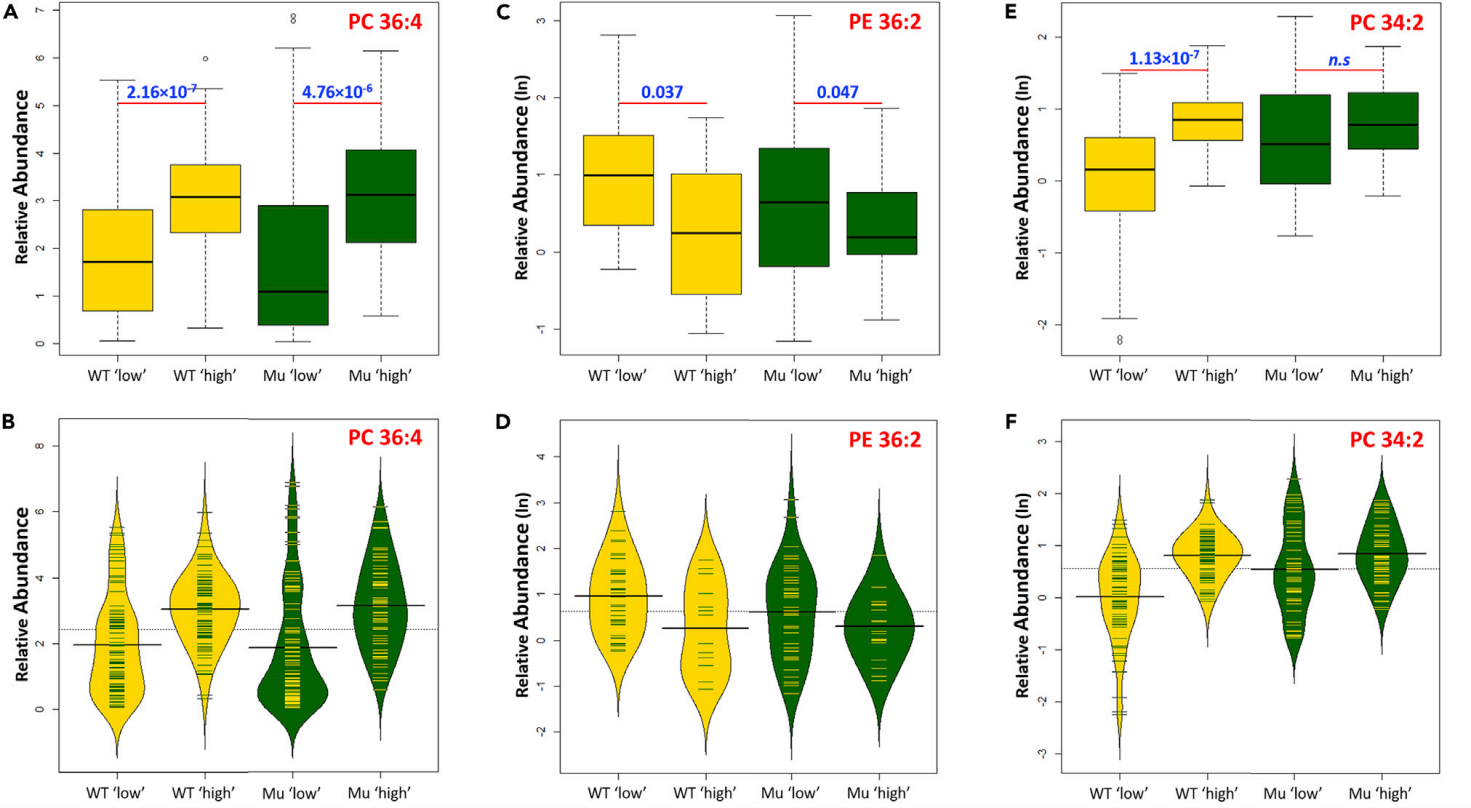
Boxplots and Beanplots Showing Lipid Abundances in Wild-Type and A53T Mutant Neurons after Stratifying Samples by Their Abundance of PC 36:2 (A) Boxplot showing the abundance of PC 36:4 in cells with high and low levels of PC 36:2; p values calculated using generalized linear model. (B) Beanplot showing the relative distribution of the abundance of PC 36:4 in cells with high and low levels of PC 36:2. (C) Boxplot showing the abundance of PE 36:2 in cells with high and low levels of PC 36:2; p values calculated using generalized linear model. (D) Beanplot showing the relative distribution of the abundance of PE 36:2 in cells with high and low levels of PC 36:2. (E) Boxplot showing the abundance of PC 34:2 in cells with high and low levels of PC 36:2; p values calculated using generalized linear model. (F) Beanplot showing the relative distribution of the abundance of PC 34:2 in cells with high and low levels of PC 36. PC, glycerophosphocholine; Mu, *SNCA-A53T* mutant dopaminergic neurons.

To further explore this heterogeneity, we stratified samples based on their abundance of PC 36:2 and assessed if the abundance of other lipids mirrored this heterogeneity. This showed that in WT nine lipids were significantly different (p < 0.05) between cells with high and low abundances of PC 36:2, whereas eight lipid variables were significant in *SNCA-A53T* dopamine neurons. Of these, six lipids were common between the genotypes, three were specific to WT, and two were specific to *SNCA-A53T* including the ratio of total PC to total PE (Table 2). The abundance of PC 36:4 in cells stratified by their PC 36:2 levels revealed similar patterns in both WT and *SNCA-A53T* populations, with dopamine neurons with high PC 36:2 having high PC 36:4 (Figure 5A). Although there were differences in the abundance and distribution of PC 36:4 in cells with high and low PC 36:2, this was similar between genotypes (Figure 5B). The same was observed when we analyzed the abundance of PE 36:2 with similar patterns observed between genotypes (Figures 5C and 5D); however, in this case neurons with more PC 36:2 have less PE 36:2. Cells with high PC 36:2 had higher levels of PC 34:2 in both genotypes; however, this difference was only significant in WT neurons (Figures 5E and 5F).

**Table 2:** Lipids with Significantly Different Abundances in Cells with High and Low Levels of PC(36:2)

	Mutant		Wild-Type	
	p Value	FC	p Value	FC
PC 34:1	0.0001	0.679	1.28 × 10^−7^	0.628
PC 34:2			1.13 × 10^−7^	0.578
PC 36:1			0.0002	0.591
PC 36:4	4.76 × 10^−6^	0.610	2.16 × 10^−7^	0.641
PC 38:4	0.0009	2.32	0.038	1.61
PC 40:3			0.0041	3.29
PE 36:1	0.0061	4.31		
PE 36:2	0.047	1.79	0.037	1.81
PE 36:3	0.0023	3.96	0.013	3.17
SM 36:1	0.0047	6.29	0.0005	3.64
PC/PE	0.011	0.36		

FC, fold change relative to cells with low PC(36:2); PC;, glycerophosphocholine; PE, glycerophosphoethanolamine; SM, sphingomyelin.

## Discussion

This platform is the first single cell approach that enables high-throughput lipid profiling of specific cell types without the need for additional imaging analysis. This coupled with a high degree of standardization and robust quality control has enabled us to apply untargeted lipid profiling to individual cells and explore the functional heterogeneity of cellular populations and the implications for disease pathology. We believe this to be a major technological advance with wide applications for biomedical research.

### Cell Location, Throughput, Quality Control, Signal Coverage

The advent of iPSCs offers the opportunity to study otherwise inaccessible cell types such as functional human neurons. However, the limitations of this model need to be acknowledged, in particular the inherent variability associated with iPSC techniques highlighted in previous studies (Volpato and Webber, 2020). To overcome this limitation, we generated isogenic *SNCA-A53T* iPSCs, a well-established approach in the field of iPSC modeling to reduce cellular variability.

As with single cell genomics and transcriptomics the requirement to analyze large numbers of cells means that these approaches need to be high throughput. In this study, we analyzed four independent replicates of two genotypes of human dopamine neurons. To capture the cellular heterogeneity of these samples we analyzed 70 cells per biological replicate leading to a total of 560 analyzed cells. Our platform enables these 560 samples to be analyzed in 2 days (including the automated data processing), underlining the high-throughput nature of this method.

We used FACS to sort TH-positive dopamine neurons into individual wells using an RFP reporter to improve the throughput and specificity of the platform. The use of the TH-RFP coupled with an appropriate gating strategy provides specificity and ensures that all cells analyzed are individual dopamine neurons. Using FACS also allowed for quick sample isolation or large number of cells without the requirement for additional imaging analysis to confirm cell numbers and identity as previously required.

This need to analyze large numbers of samples requires a high-throughput platform with robust quality control strategies to ensure the quality of the data generated. In the development of this platform we automated as many processes as possible, to both maximize the number of samples that can be analyzed in a given time and to standardize analysis to minimize sources of analytical variation. With cells being sorted onto all seven analytical plates at the same time and stored at −80°C before analysis it was important to ensure that storage was not effecting the lipid composition of the cells. Although in the raw data batches effects were observed between plates, normalization of the data removed these effects with no plate to plate differences seen in either QC or single cell samples at either the univariate or multivariate level demonstrating that sample storage is not introducing any bias into the lipid composition of the cells. In the normalized data we also showed that there was no relationship between injection order and lipid profile composition (Figure S1) demonstrating that the data generated from the first and last samples are comparable.

It is crucial to control for cell size as this is the most important factor controlling the measured lipid abundance, as a larger cell will have greater total signal abundance because it contains more lipids. However, as cell size will also determine the total lipid signal observed, normalizing lipid abundances to the total signal controls for the size of the cell analyzed.

For this method we used LESA as it requires lower solvent volume than traditional extraction protocols producing more concentrated extracts. However, when using LESA-MS-based approaches for single cell lipid profiling locating the cell for analysis is a significant challenge. Although other methods have used imaging approaches to locate the cell for analysis (Ellis et al. 2012; Neumann et al., 2019), this is time consuming and would significantly reduce the throughput of the method. Instead we calibrated the FACS to maximize the chance of a cell being dispensed in the center of the well, with the LESA analysis subsequently performed on that spot; however, in some cases the LESA missed the cell. We identified these failed analysis by searching the generated spectra for signals from the two most abundant lipids (PC 34:1 and PC 36:2), and if neither of these were present then the sample was considered a failure and excluded from subsequent analysis. It is also important to identify and exclude samples where two cells had been erroneously dispensed into the same well. We did this by looking for samples that had significantly higher abundance of all the lipids measured (as an analysis of two cells will increase the amount of all lipids in the well relative to a single cell), and we excluded samples from further analysis if 80% of lipids had an abundance 2.5 standard deviations above the mean.

As in traditional lipidomic approaches we used signal to noise, mass accuracy (ppm), and missing value thresholds to identify lipid signals, although we applied more relaxed cutoffs for each of these parameters than are used in normal lipidomic methods (Harshfield et al., 2019). The lower signal abundance achieved when analyzing a single cell means that signal-to-noise ratios will be lower than when analyzing bulk cell pellets, tissue, or bio-fluid samples. This low intensity does also leads to slightly lower mass accuracy and higher percentage of samples in which a lipid will fall between the lower limit of detection, which means that if we applied the standard values for these threshold real lipid signals would have been missed.

For signal identification, although the QC cutoffs we applied are less stringent than in traditional lipidomics approaches they are more stringent than those used in other single cell lipid profiling methods. For example, whereas we required a signal-to-noise threshold of >1.5, mass accuracy of <10 ppm, r > 0.5 in a cell “standard” curve, and a signal to be present in at least 10% of analyzed cells with many of the identifications validated across our multiple experiments, Neumann et al. (2019) did not apply any signal to noise, mass accuracy, or cell “standard” curve cutoffs and included lipids measured in less than 1% of cells.

After optimization and quality control we measured 37 lipids (Table 1), which is lower than reported in this previous study; however, this is a result of our quality controls being more stringent to allow meaningful comparison of the generated data. Although the number of lipids reported here is lower than for a traditional lipidomics study (using tissue or plasma, Ellis et al., 2012; Neumann et al., 2019; Thiele et al., 2019), it provides sufficient coverage to interrogate the lipid metabolism of a single cell. When we look at the abundances of lipids measured from a bulk cell sample (Figure S5) it can be seen that the distribution is not linear, and unsurprisingly it can also be seen that the lipids measured in a single cell are generally the lipids that are the most abundant in the bulk samples (Figure S5). Potentially of more interest is the fact that just below the lower limit of detection (LLOD) in the single cell method there are a lot of lipids within a narrow range of abundances, suggesting that a small increase in the sensitivity of the method will likely lead to a large increase in the number of lipids measured. Despite current limitations, developments in mass spectrometric instruments' sensitivity should soon provide technical improvements to expand this platform to a larger number of detectable lipids.

### Advantages of Single Cell over “Population” Lipid Profiling Approaches

When we compared the “population” and single cell lipid profiles we observed differences between WT and *SNCA-A53T* mutant dopamine neurons driven predominantly by PC 36:2. However, only by single cell analysis we were able to identify an overlap between the two genotypes both in the multivariate and univariate analyses (Figure 4). This shows that the population level analysis failed to capture the heterogeneity of the biological processes at work in human dopamine neurons. There was also significant overlap in the abundance of PC 36:2 (Figure 4D) with the WT cells showing a much more even distribution abundance across their concentration range (Figures 4E and 4F) with a high number of *SNCA-A53T* neurons presenting with low PC 36:2. These results highlight the need to develop and apply methods for single cell analysis to capture both the heterogeneity of the model in study and important cell-type-specific differences. This is further supported by our recent single cell transcriptomic analysis of this model that leads to the identification and characterization of cellular heterogeneity, which would not be possible using traditional bulk approaches (Fernandes et al., 2020).

### Impaired Membrane Function in *SNCA-A53T* Dopamine Neurons

PCs are key components of the cell membrane (van Meer et al., 2008), and changes in the balance of species of different chain lengths as well as the PC to PE ratio (van der Veen et al., 2017) affect membrane fluidity, structure, and function (van der Veen et al., 2017; Paris et al., 2010; Sciacca et al., 2012). Several studies have suggested that *SNCA* plays a role in lipid transport and synaptic membrane biogenesis (Jo et al., 2000), so the *SNCA-A53T* mutation could result in changes in the composition of the cell membrane and thus potential impairment of synaptic transmission. The results of the “population” lipid profiling show higher levels of PC 36:2 in all WT replicates with no overlap between the genotypes (Figure 4B). This suggests that the A53T mutation is causing membrane remodeling to occur evenly across all cells, potentially resulting in a distinct cellular morphology not present in the WT neurons.

After stratifying lipid abundances based on PC 36:2 11 lipid variables were significantly different between cells with high and low abundance of this lipid. Of these, six were common to both genotypes, and with four lipids differing significantly between WT and A53T mutant dopamine neurons multiple factors appear to be controlling the lipid composition of a cell. Therefore, single cell approaches provide an opportunity to explore how the A53T mutation interacts with these factors to modulate lipid metabolism. The ratio of total PC to total PE in the cell membrane has been shown to have major effects on the fluidity of the bilayer and vesicle formation as well as the composition and prevalence of transmembrane proteins (Li et al., 2006; Brown and Seelig 1978). Thus it is interesting that in *SNCA-A53T* dopamine neurons we see a larger difference in the PC:PE ratio between cells with high and low PC 36:2 levels than is observed in the WT (Table 2). The majority of the mutant cells have low PC 36:2, resulting in low PC:PE ratios for a higher proportion of these cells.

Healthy cells have a range of mechanisms to respond to normal physiological stimuli as well as to adapt to stress. In this study we have shown a greater heterogeneity in lipid composition in WT neurons, and given that the cell membrane is the largest source of lipids in the cell it is likely that this reflects heterogeneity in the makeup of the cell membrane. It is plausible that this heterogeneity in membrane composition is integral to the mechanisms by which cell populations respond to membrane stress and the skewed distribution in the mutant neurons could impair their ability to respond to stimuli (Fu et al., 2011; Hannun and Luberto, 2000).

### Strengths of Study

#### High Throughput

Single cell approaches require the analysis of large numbers of cells to capture inter-cell heterogeneity. This results in the requirement of a large number of cells from each biological replicate, significantly increasing the number of analysis required per experiment. Our method is capable of measuring the lipid profile of 280 samples in 24 h meaning that in just 2 days we were able to analyze sufficient samples to identify different distributions of lipid abundance between WT and *SNCA-A53T* dopamine neurons.

#### Cell-Type Specificity

Tissue and cell culture models are heterogeneous and made up of diverse cell types in different proportions, which can make single cell analysis difficult. Previous lipidomics methods have coupled with imaging approaches to identify the type of cell being analyzed. Although this approach is useful for some applications, it is not translated into a high-throughput platform capable of analyzing large number of samples required to address questions of cellular heterogeneity. Our approach of sorting cells using FACS before analysis ensures that we are specifically analyzing a single and specific cell type of interest. This means that differences detected between generated lipid profiles are the result of heterogeneity within a specific cell population rather than differences between the compositions of different cell types.

#### Quality Control

The need to analyze large numbers of samples in single cell approaches means that robust quality control procedures need to be implemented to ensure that all the generated data is comparable. Although some studies have measured lipids in single cells (Ellis et al., 2012; Neumann et al., 2019; Thiele et al., 2019; Standke et al., 2019) and some have measured in large numbers of cells (Neumann et al., 2019), these approaches have incorporated limited quality control procedures to ensure data consistency across sample sets. In this study we also included a pooled quality control corresponding to unsorted cells, which was added to each plate and enabled us to correct for inter-plate variability (Figure 3). This enabled us to perform large-scale experiments while accounting for possible sources of analytical variance that could confound our results.

#### Validation of Signal Identification

Signal identification in single cell lipid profile data is challenging because the majority of signals are low abundance and are often not measured in a large proportion of samples making it difficult to distinguish real signals from analytical noise. To avoid reporting false-positives in this study we analyzed TH-positive dopamine neurons from three independent experiments to identify lipids that were measured consistently. To provide clarity we have defined three levels of confidence in our putative assignments with species identified in all three experiments given our highest confidence (Level 1) and signals only putatively identified in one experiment given our lowest confidence (Level 3).

In conclusion, this work provides a method for high-throughput lipid profiling at the single cell level. As a proof of principle, we used this platform to profile individual human dopamine neurons resulting in the identification of lipid alterations that will further our understanding of PD pathology. This technological advance potentially has major applications across a wide range of biomedical research fields, allowing the opportunity to explore and overcome metabolic heterogeneity in complex cellular populations.

### Limitations of Study

#### Putative Lipid Assignment

Ideally glycerophospholipids would be identified as their lipid head group and the specific pair of fatty acid side chains rather than by the head group and the total number of carbons and double bonds, e.g., PC 16:1/18:0 rather than PC 34:1. This additional structural detail can improve biological interpretation as the different fatty acid configurations can have differing biological functions. This additional structural detail is usually obtained by fragmenting the parent molecule. However, it is extremely difficult to obtain clean fragmentation spectra from low-abundance signals, making it impossible to obtain this detailed structural information in current single cell approaches. It is for this reason that other single cell approaches have also only been able to annotate lipids to the level of the total number of carbons and double bonds. However, in the future we hope that the availability of more sensitive mass spectrometers will allow us to generate fragmentation spectra and obtain these more detailed annotations.

#### Coverage of the Lipidome

Lipids represent the largest and most diverse group of metabolites in the human body consisting of dozens of structural classes and potentially thousands of structurally distinct molecules. In this study we have identified 81 lipid species from six lipid classes, which is only a small proportion of the total lipids present. However, if we look at the distribution of the abundance of the identified lipids in a lipid dataset generated from a bulk cell pellet it can be seen (Figure S5) that the assigned lipids are the most abundant lipids. It can clearly be seen that the distribution of lipid abundances is not linear, so even a small improvement in the lower limit of detection can potentially greatly improve the coverage of the lipidome. This improvement can potentially be achieved by using more sensitive mass spectrometry equipment in the future.

#### Exclusion of Internal Standards

Traditional lipidomics methods include internal standards to correct for analytical variation within the data. However, internal standards only work when the amount of lipid in a sample is known, as analytical variation will then be the major source of stochastic variance. If we consider single cell approaches, each sample will consist of one cell, yet the amount of total lipid will differ between samples due to differences in cell size. This means that the intensity of the lipid signals will be independent of the intensity of the internal standards. To support this, we showed that lipid and internal standard signal correlated in plasma samples but did not in single cell samples (Figure S6).

#### Missing Data

In an ideal scenario all the lipids detected would be measured in all the samples analyzed; however, this is never the case as across samples some lipids will fall below the lower limit of detection. Large numbers of missing values can be problematic in both multivariate and univariate statistical analysis by potentially obscuring the relationship of lipid abundances. This is particularly an issue in single cell approaches where low and medium abundance lipids will only be measured in larger cells. Improving the sensitivity of the platform and lowering the LLOD will increase the proportion of samples that we can measure of a given lipid in helping to alleviate this problem.

### Resource Availability

#### Lead Contact

Further information and requests for resources and reagents should be directed to the lead contact Albert Koulman (ak675@medschl.cam.ac.uk).

#### Material Availability

This study did not generate any unique reagents.

#### Data and Code Availability

All the code used in this study is shown in the Supplemental Information of the article.

## Methods

All methods can be found in the accompanying Transparent Methods supplemental file.

## Supplemental Information

Document S1. Transparent Methods, Figures S1–S10, Tables S1 and S2, and Schemes S1 and S2

Table S3. List of 484 Lipid Species Used to Interrogate Single Cell Spectra, Related to Transparent Methods CE, cholesteryl ester; Cer, ceramide; DG, diglyceride; PC, glycerophosphocholine; PE, glycerophosphoethanolamine; PS, glycerophophoserine; SM, sphingomyelin; TG, triglyceride.

## Footnotes

Supplemental Information can be found online at https://doi.org/10.1016/j.isci.2020.101703.
