# Supplementary material for: Development and Application of High-Throughput Single Cell Lipid Profiling: A Study of SNCA-A53T Human Dopamine Neurons
Source: iScience. 2020 Oct 21;23(11):101703. doi: 10.1016/j.isci.2020.101703 (PMC7644967; doi:10.1016/j.isci.2020.101703)
Supplement: Document S1. Transparent Methods, Figures S1–S10, Tables S1 and S2, and Schemes S1 and S2 [file mmc1.pdf]

iScience, Volume *23*

Supplemental Information

Development and Application of High-Throughput Single Cell Lipid Profiling: A Study of *SNCA-A53T* Human Dopamine Neurons

Stuart G. Snowden, Hugo J.R. Fernandes, Josh Kent, Stefanie Foskolou, Peri Tate, Sarah F. Field, Emmanouil Metzakopian, and Albert Koulman

Supplemental Figures

Supplemental Figure 1 PCA Scores plots showing the compositional relationship between cells from the same population and their relative injection order. Related to Figure 3.

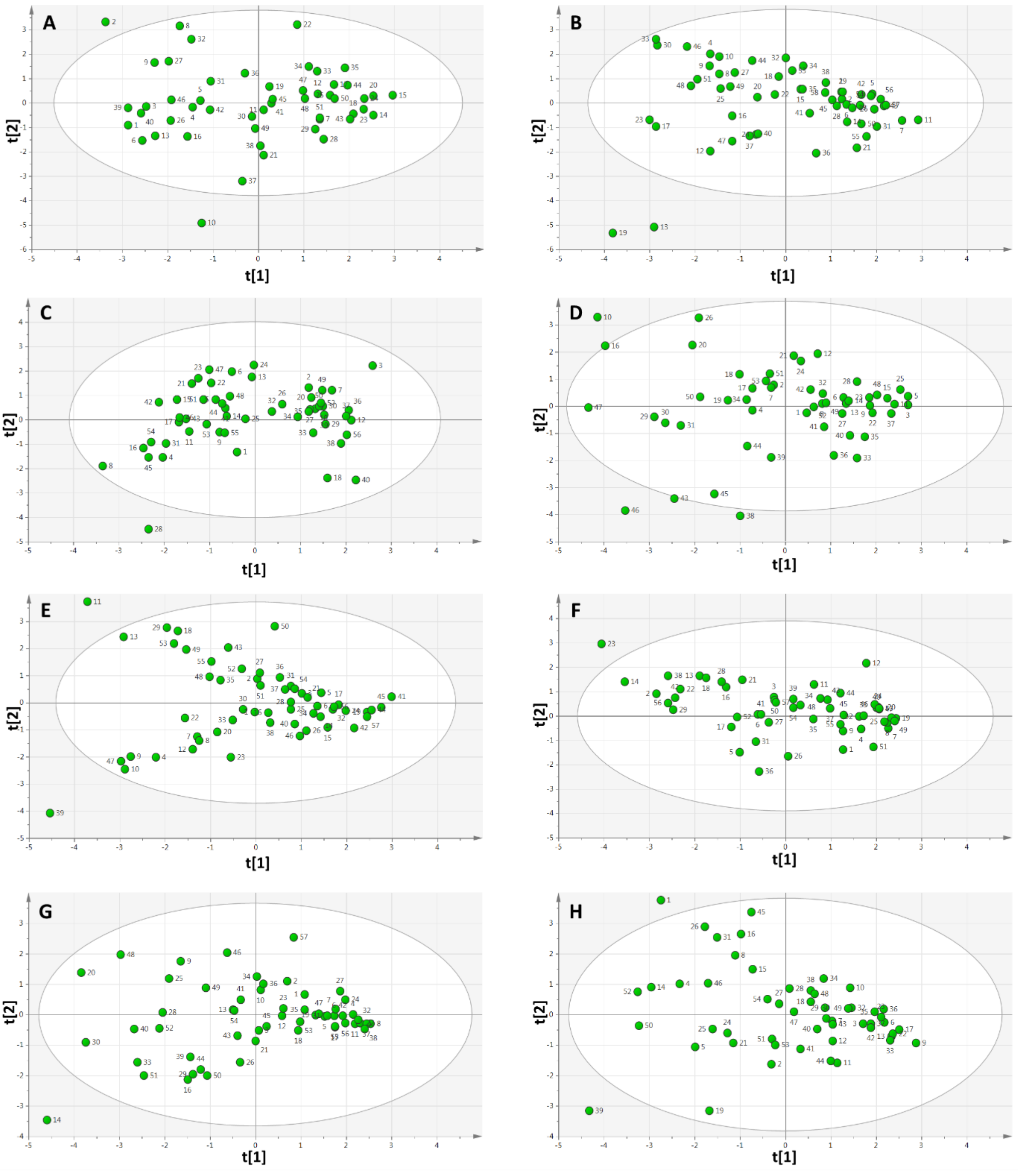

A) Wild type 1 B) Wild type 2 C) Wild type 3 D) Wild type 4 E) *SNCA-A53T* 1 F) *SNCA-A53T* 2 G) *SNCA-A53T* 3 H) *SNCA-A53T 4*

Supplemental Figure 2 Plots comparing the abundance of PC 36:1 in wild type and A53T mutant cells. Related to Figure 4.

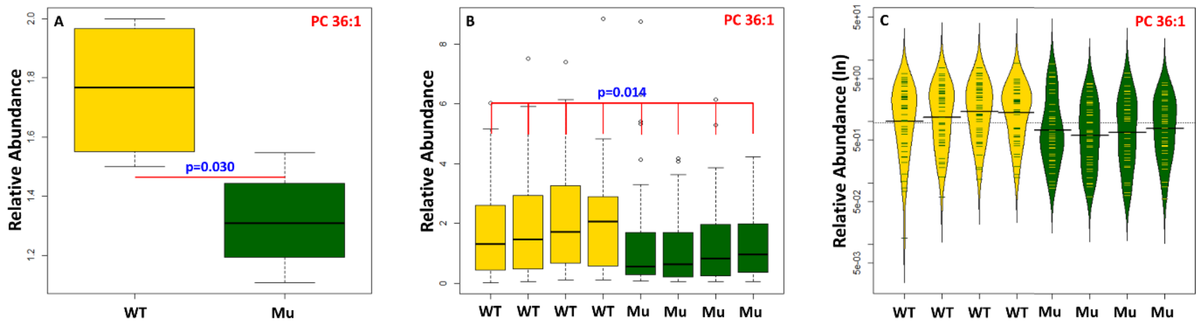

A) Boxplot comparing the abundance of wild type and mutant cells (Mu) in ‘population’ level data B) Boxplot comparing the abundance in individual wild type and mutant cells (Mu) populations using single cell data, p-value calculated using generalised linear models C) Beanplot comparing the distribution of abundances in individual wild type and mutant cell (Mu) populations using single cell data. Mu*;* *SNCA-A53T* mutant dopaminergic neurons.

Supplemental Figure 3 Plots comparing the abundance of PC 34:2 in wild type and A53T mutant cells. Related to Figure 4.

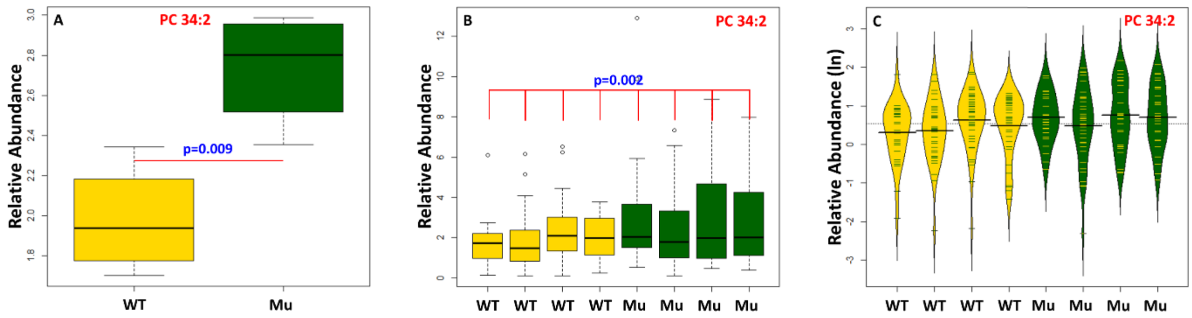

A) Boxplot comparing the abundance of wild type and mutant cells (Mu) in ‘population’ level data B) Boxplot comparing the abundance in individual wild type and mutant cell (Mu) populations using single cell data, p-value calculated using generalised linear models C) Beanplot comparing the distribution of abundances in individual wild type and mutant cell (Mu) populations using single cell data. Mu; *SNCA-A53T* mutant dopaminergic neurons.

Supplemental Figure 4 Plots comparing the abundance of PC 32:0 in wild type and A53T mutant cells. Related to Figure 4.

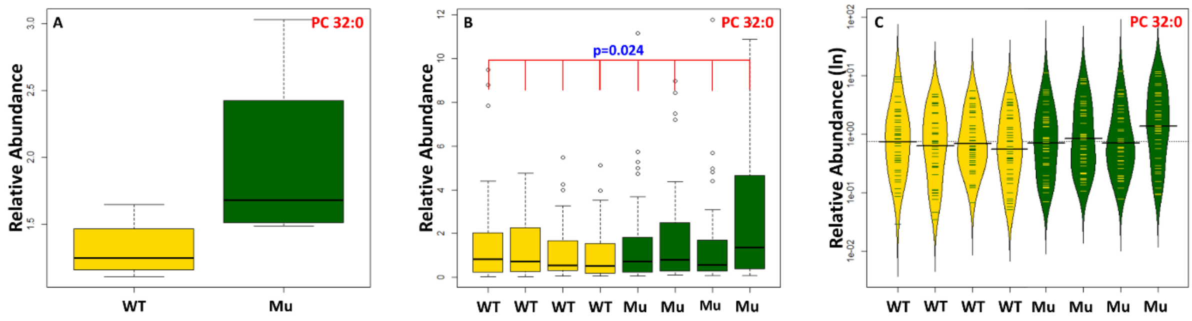

A) Boxplot comparing the abundance of wild type and mutant cells (Mu) in ‘population’ level data B) Boxplot comparing the abundance in individual wild type and mutant cell (Mu) populations using single cell data, p-value calculated using generalised linear models C) Beanplot comparing the distribution of abundances in

individual wild type and mutant cell (Mu) populations using single cell data. Mu; *SNCA-A53T* mutant dopaminergic neurons.

Supplemental Figure 5 Scatter plot showing the relative abundance of level 1,2 and 3 single cell annotations in a bulk lipidomics dataset generated from the same cell lines. Related to Figure 3.

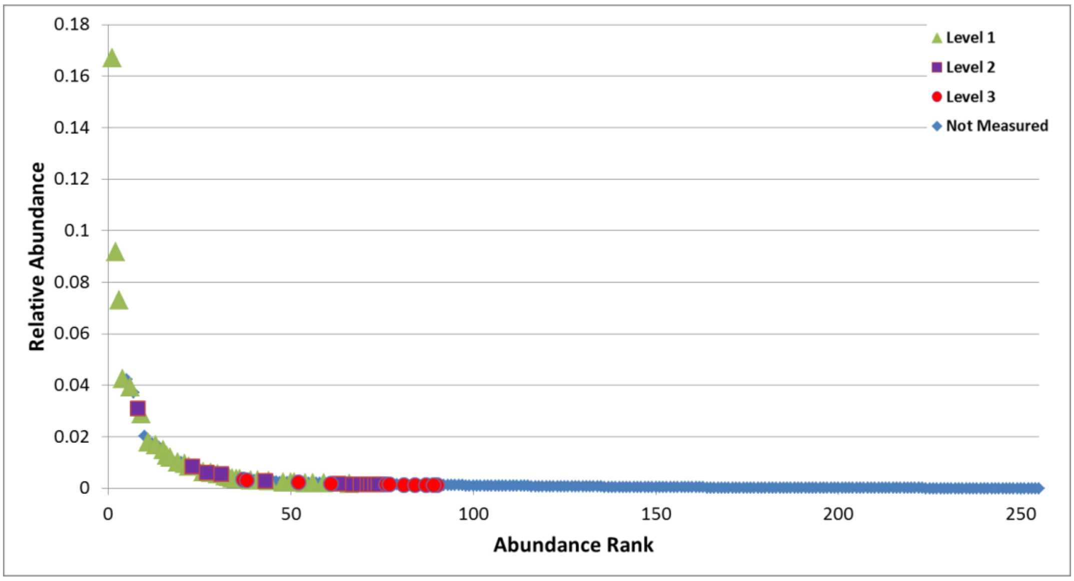

Supplemental Figure 6 Scatter plots showing the relationship between the abundance of PC internal standard and the abundance of PC 34:1 in both plasma and single cell samples. Related to Figure 3.

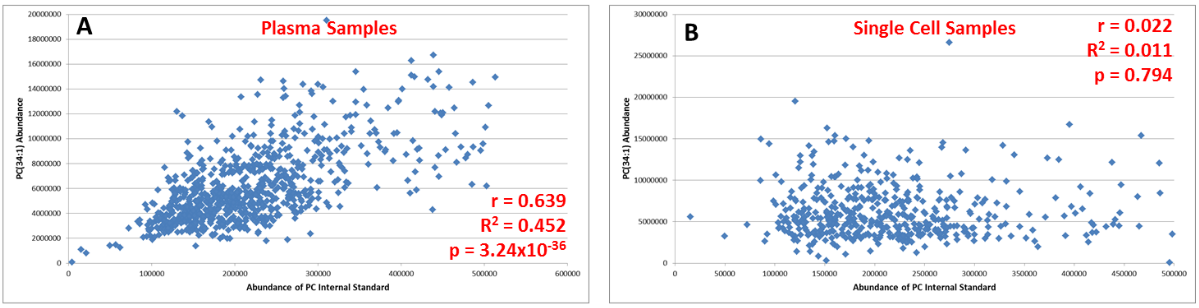

p-values calculated using generalised linear models. PC; glycerophosphocholine.

Supplemental Figure 7 Plots of the signal abundance and signal to noise ratio of PC C16:0-d31/C18:1 when optimising infusion back pressure. Related to Figure 3.

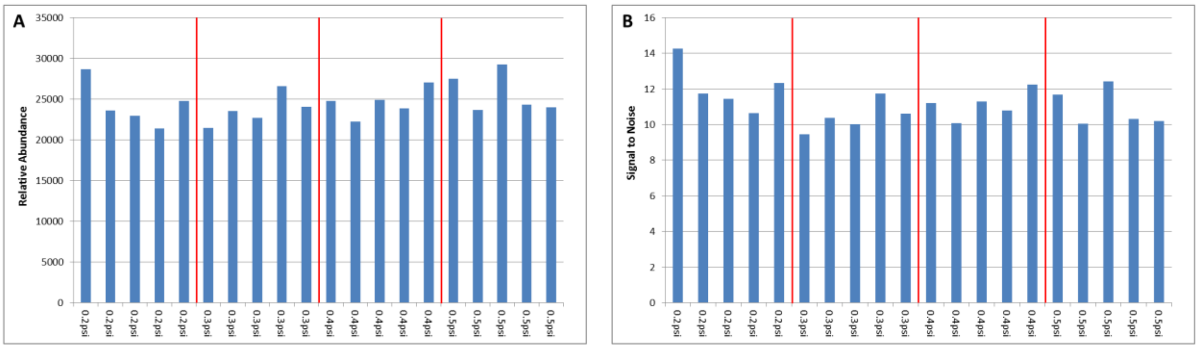

A) signal abundance in individual test samples B) signal to noise in individual test samples

Supplemental Figure 8 Plots of the signal abundance and signal to noise ratio of PC C16:0-d31/C18:1 when optimising electrospray ionisation current. Related to Figure 3.

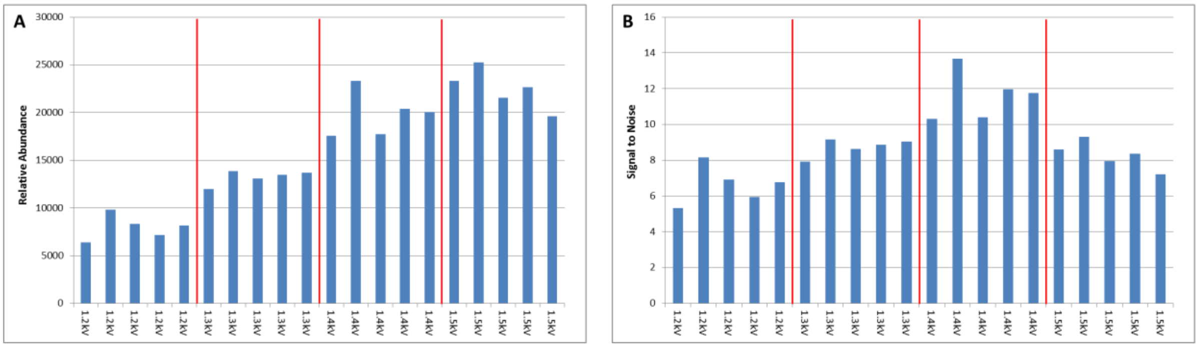

A) signal abundance in individual test samples B) signal to noise in individual test samples

Supplemental Figure 9 Plots of the signal abundance and signal to noise ratio of PC C16:0-d31/C18:1 when optimising the mass window analysed. Related to Figure 3.

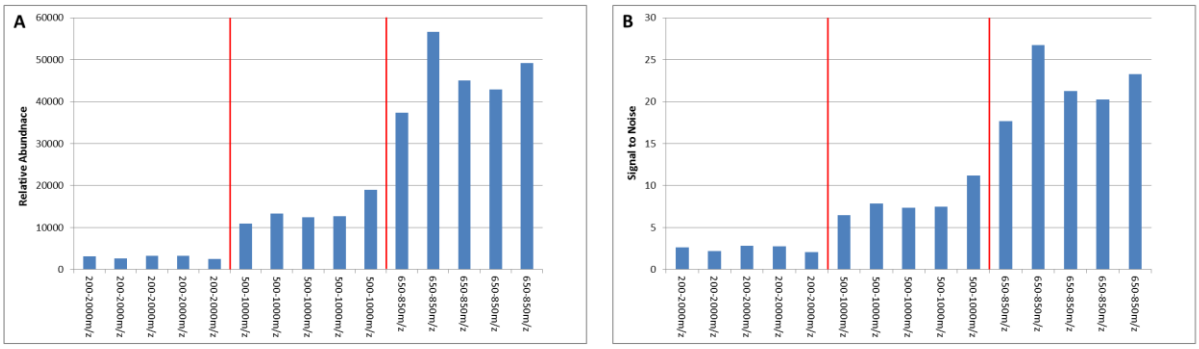

A) signal abundance in individual test samples B) signal to noise in individual test samples

Supplemental Figure 10 Plots of the signal abundance and signal to noise ratio of PC C16:0-d31/C18:1 when optimising the solvent volume used for LESA. Related to Figure 3.

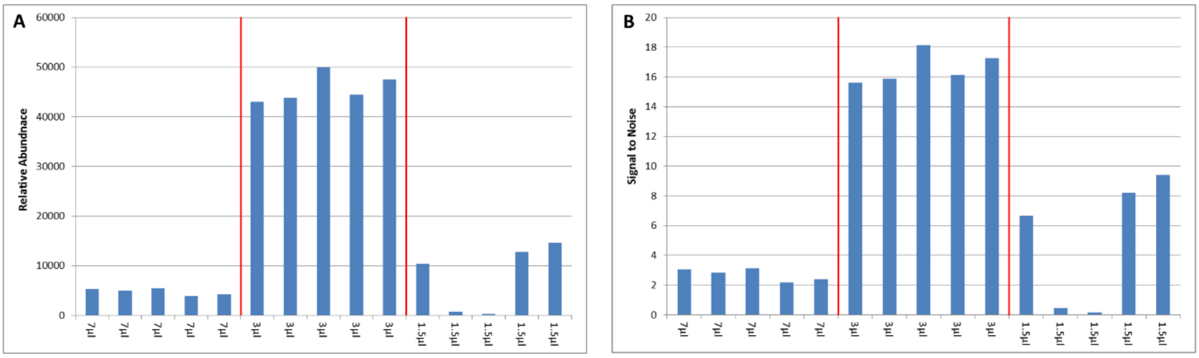

A) signal abundance in individual test samples B) signal to noise in individual test samples

Supplemental scheme

Supplemental scheme 1 R script used to run our in-house pipeline for comparing generated spectra to list of know lipids. Related to Table 1.

```
source("hrms.R")
files = list.files(".", pattern=".mzXML")
system.time(
  for (i in 1:length(files)) {
    main(files[i],rtwin=c(20,78),mzwin=c(650,850))
  }
)
results <- signals_deviations()
```

Supplemental Scheme 2 R script contained in "hrms.R" referred to in supplemental scheme 1 that compares generated spectra to list of known lipids listed in Supplemental table 3. Related to Table 1.

```
main <- function(filename,rtwin,mzwin) {
  require(xcms)
  require(data.table)
  targets <- read.table("./LipidList.csv" , header=T, sep=',')
  targets <- data.table(targets)
  options("nwarnings" = (length(targets$mz)+50)) # we need to get at least as many warnings as
targets
  spectrum <- getspectra(filename=filename, rt=rtwin, mz=mzwin)
  tgts <- peaktable(targets,spectrum)
  write.csv(tgts, file = gsub(pattern=".mzXML", x=filename, replacement=".csv"), row.names=F)
}

signals_deviations <- function() { # the csv files must be in the active directory
  x <- read.csv(gsub(pattern=".mzXML", x=files[1], replacement=".csv"),header=T)
  targets <- read.table("./LipidList.csv" , header=T, sep=',')
  targets <- data.table(targets)
  signals <- data.frame(x$targets.name,targets$mz)
  for (i in 1:length(files)) {
    x <- read.csv(gsub(pattern=".mzXML", x=files[i], replacement=".csv"),header=T)
    signals[files[i]] <- data.frame(x$signal)
  }

  x <- read.csv(gsub(pattern=".mzXML", x=files[1], replacement=".csv"),header=T)
  deviations <- data.frame(x$targets.name,targets$mz)
  for (i in 1:length(files)) {
    x <- read.csv(gsub(pattern=".mzXML", x=files[i], replacement=".csv"),header=T)
    deviations[files[i]] <- data.frame(x$mz_deviation)
  }
  results <- list(signals,deviations)
  write.csv(results[[1]],file="signals.csv", row.names=F)
  write.csv(results[[2]],file="deviations.csv", row.names=F)
  return(results)
}

getspectra <- function(filename,rt,mz) {
  spectra <- list()
```

```
  spectrum <- getSpec(xcmsRaw(filename), rtrange=rt, mzrange=mz)
  spectrum[,"mz"] <- round(spectrum[,"mz"], digits=4)
  spectrum <- as.data.table(spectrum)
  spectrum <- spectrum[,mean(intensity), by=mz]
  spectrum <- na.omit(spectrum)
  setkey(spectrum,mz)
  return(spectrum)
}

peaktable <- function(targets,spectra) {
  nearest_mz <- vector(length=length(targets$mz)) #predefine length later
  signal <- vector(length=length(targets$mz)) #predefine length later
  for (i in 1:length(targets$mz)) {
    target <- targets[i,mz]
    peak <- peakfind_midpoint(target,spectra,0.01,warnings)
    nearest_mz[i]<-peak[1,mz]
    signal[i]<-peak[1,V1]
  }

  mz_deviation <- targets[,mz] - nearest_mz
  peak_id <- data.frame(targets$name,targets$mz,nearest_mz,mz_deviation,signal)
  return(peak_id)
}

peakfind_midpoint <- function(target,spectra,hwidth,warnings) {
  window <- subset(spectra, spectra$mz > target-hwidth & spectra$mz < target+hwidth)
  if (nrow(window)==0) { # no data for target?
    peak = data.table('mz'=target,'V1'=0) # enter zero intensity for target mass
  } else if (sum(window$V1) < 5000) { # very low s/n?
    peak <- peakfind_max(target,spectra,hwidth) # uses older peakmax finder for low s/n peaks, while
less accurate this helps with exception handling dramatically
    warning(paste("low signal/noise found for target mass-", target, "-using older peakmax finder.
Identification may not be accurate", sep=" "))
  } else { # now we run the peak width peak finder
    peak <- peakfind_max(target,spectra,hwidth)
    hh_close=peak[1,V1]/2
    window <- subset(spectra, spectra$mz > peak$mz-hwidth & spectra$mz < peak$mz+hwidth)
    if (window$V1[length(window$mz)] > hh_close | window$V1[1] > hh_close) { # window doesn't
sample the width of the peak?
      setkey(window,V1) #this will sort table by intensity, thus finding peak maximum as last entry in
table
      peak <- window[length(window$mz)] #get last entry of table for the peak maximum
      window <- subset(spectra, spectra$mz > peak$mz-hwidth & spectra$mz < peak$mz+hwidth)
      if (window$V1[length(window$mz)] > hh_close | window$V1[1] > hh_close) { # is the bad
sampling of peak due to interference?
        setkey(window,V1) #this will sort table by intensity, thus finding peak maximum as last entry in
table
        peak <- window[length(window$mz)] #get last entry of table for the peak maximum
        warning((paste("interfered peak detected for target mass-", target, "-older peak_max() function
used", sep=" ")))
      }
    } else {
      ## for resolved peaks (at half height) the follow code is run ##
      setkey(window,V1) #this will sort table by intensity, thus finding peak maximum as last entry in
table
      peak <- window[length(window$mz)] #get last entry of table for the peak maximum
```

```
      hh=peak[1,V1]/2
      nearmz=peak[1,mz]
      left_mzs <- c(max(subset(window, window$mz < nearmz & window$V1 <
hh)$mz),min(subset(window, window$mz < nearmz & window$V1 > hh)$mz))
      left_int <- c(max(subset(window, window$mz < nearmz & window$V1 <
hh)$V1),min(subset(window, window$mz < nearmz & window$V1 > hh)$V1))
      right_mzs <- c(max(subset(window, window$mz > nearmz & window$V1 <
hh)$mz),min(subset(window, window$mz > nearmz & window$V1 > hh)$mz))
      right_int <- c(max(subset(window, window$mz > nearmz & window$V1 <
hh)$V1),min(subset(window, window$mz > nearmz & window$V1 > hh)$V1))
      midpoints <- data.frame(left_mzs,left_int,right_mzs,right_mzs)
      ### there has got to be a way to combine the last five rows into one row
      coordinates <- list()
      left <- coefficients(lm(left_int ~ left_mzs, data=midpoints))
      right <- coefficients(lm(right_int ~ right_mzs, data=midpoints))
      midpoint <- ((hh-left[1])/left[2]+(hh-right[1])/right[2])/2 # midpoint between the intersection
points of both lines from a y=hh flat line
      peak$mz <- round(midpoint[1], digits=4) # modify the peaks variable with the new more accurate
m/z value
    }
  }
  return(peak)
}

peakfind_max <- function(target,spectra,hwidth) {
  window <- subset(spectra, spectra$mz > target-hwidth & spectra$mz < target+hwidth)
  setkey(window,V1) #this will sort table by intensity, thus finding peak maximum as last entry in table
  peak <- window[length(window$mz)] #get last entry of table for the peak maximum
  #plot(window, type='h', lwd=1)
  return(peak)
}

if(!interactive()){
  args <- commandArgs(trailingOnly = TRUE)
  f <- args[1]
  main(f,rtwin=c(85,130),mzwin=c(190,1800))
}
```

Supplemental Tables

Supplemental Table 1 comparison of signal abundance and mass deviation of individual lipids in both extraction blanks and single cell samples. Related to Figure 1.

	Abundance			Deviation		
	**Blanks**	**Samples**	**p-value**	**Blanks**	**Samples**	**p-value**
**PC 30:1**	42	803	3.1×10^{-4}			
**PC 32:0**	70	3117	5.1×10^{-10}	8.6	2.2	3.0×10^{-10}
**PC 32:1**	633	7122	0.003			
**PC 32:2**	215	651	0.001			
**PC 32:4**	80	232	0.01			
**PC 34:0**	5	625	4.1×10^{-6}			
**PC 34:1**	614	10330	4.6×10^{-13}	14.7	1.8	4.6×10^{-13}
**PC 34:2**	97	2977	7.1×10^{-32}	8.3	2.1	2.1×10^{-9}
**PC 34:3**	309	1445	8.7×10^{-12}			
**PC 34:4**	71	1575	4.5×10^{-34}	6.3	8.4	0.03
**PC 36:1**	160	2062	3.8×10^{-7}	11.5	2.6	9.7×10^{-11}
**PC 36:2**	75	2619	1.2×10^{-46}	10.8	3.0	9.9×10^{-15}
**PC 36:3**	124	1945	9.1×10^{-38}	9.3	8.1	0.02
**PC 36:4**	400	3541	1.2×10^{-8}	16.2	5.1	3.8×10^{-11}
**PC 36:5**	96	2295	0.07	13.3	6.2	0.05
**PC 38:3**	41	294	1.9×10^{-9}			
**PC 38:4**	56	179	1.7×10^{-6}	7.4	7.1	0.50
**PC 38:5**	33	792	2.2×10^{-20}			
**PC 38:8**	3	357	0.03			
**PC 40:3**	26	371	4.8×10^{-6}	6.7	4.4	0.01
**PC 40:4**	328	686	0.03			
**PC 40:5**	11	263	4.5×10^{-6}			
**PC 40:6**	826	1249	0.04			
**PC 40:9**	6	322	0.02			
**PE 34:0**	64	229	0.02			
**PE 34:1**	8	553	4.3×10^{-5}	11.3	4.6	2.1×10^{-7}
**PE 34:2**	53	510	0.01	7.2	1.9	8.4×10^{-7}
**PE 34:3**	67	235	0.04	8.1	5.7	0.04
**PE 36:0**	42	347	0.06			
**PE 36:1**	1030	2365	0.03	7.4	3.4	1.2×10^{-5}
**PE 36:3**	39	104	0.11	8.6	6.4	0.01
**PE 36:4**	39	129	0.0002	8.5	5.4	0.003
**PE 38:6**	61	154	0.006			
**SM 34:1**	66	754	0.0009	7.8	1.3	3.2×10^{-11}
**SM 36:1**	48	323	5.6×10^{-6}	7.8	1.4	1.1×10^{-8}
**SM 36:2**	135	294	0.04			

PC; glycerophophocholine, PE; glycerophosphoethanolamine, SM; sphingomyelin.

Supplemental Table 2 comparison of signal abundance and mass deviation of individual lipids in both extraction blanks and single cell samples. Related to Table 1.

	Experiment 1	Experiment 2	Experiment 3
Cer 42:1 **(Lv2)**	33.3	18.8	
Cer 44:2 **(Lv2)**		23.7	37.8
DG 42:2 **(Lv2)**	11.1	12.5	
PC 30:0 **(Lv2)**		60.0	40.5
PC 34:5 **(Lv3)**		92.5	
PC 36:0 **(Lv2)**	55.5		17.3
PC 38:2 **(Lv2)**		38.8	32.9
PC 38:6 **(Lv2)**	22.2		47.3
PC 38:7 **(Lv3)**			36.5
PC 40:7 **(Lv3)**	33.3		
PC 40:8 **(Lv3)**	22.2		
PC-O 32:0 **(Lv3)**			10.8
PC-O 34:1 **(Lv2)**		30.0	17.6
PC-O 34:2 **(Lv2)**	55.5		19.4
PC-O 36:3 **(Lv3)**		7.3	
PC-O 36:4 **(Lv2)**		13.8	19.4
PC-O 36:5 **(Lv3)**	22.2		
PC-P 34:4 **(Lv3)**	33.3		
PE 32:2 **(Lv2)**		6.3	11.9
PE 38:2 **(Lv3)**			26.1
PE 38:3 **(Lv2)**		11.7	20.4
PE 38:4 **(Lv3)**			4.3
PE 38:5 **(Lv3)**			49.8
PE 40:4 **(Lv2)**	22.2	6.3	
PE 40:5 **(Lv3)**			23.4
PE 40:6 **(Lv3)**			14.6
PE 40:7 **(Lv2)**	33.3		9.9
PE-O 34:2 **(Lv3)**			22.1
PE-O 36:1 **(Lv3)**			6.8
PE-O 36:2 **(Lv2)**	22.2		43.2
PE-O 36:3 **(Lv3)**			13.3
PE-O 36:4 **(Lv2)**		28.8	15.8
PE-O 36:5 **(Lv2)**		1.3	7.9
PE-O 38:2 **(Lv2)**	11.1	10.0	
PE-O 38:4 **(Lv3)**			10.6
PE-O 38:5 **(Lv3)**	55.5		
PE-O 38:6 **(Lv3)**			1.8
PE-O 40:5 **(Lv3)**			12.4
PE-O 40:6 **(Lv2)**		41.3	27.3
PE-P 40:7 **(Lv2)**	11.1		49.3
PS 38:5 **(Lv2)**		23.7	51.4
PS 40:4 **(Lv3)**			35.6
PS 40:6 **(Lv2)**		20.0	27.0
SM 34:0 **(Lv2)**	11.1		11.5
TG 42:4 **(Lv2)**		18.8	13.3

Cer; ceramide, DG; diglyceride, PC; glycerophophocholine, PE; glycerophosphoethanolamine, PS; glycerophophoserine SM; sphingomyelin, TG; triglyceride.

Transparent Methods

Key resources Table

Reagent or Resource	Source	Identifier
**Biological Samples**		
KOLF-2 iPSC (WT)	HipSci	RRID:CVCL_9S58
KOLF-2 iPSC (*SNCA-A53T*)	Fernandes et al, 2020	
**Chemicals and consumables**		
Isopropanol	Sigma Aldrich	Cat# I9516-1L
Methanol	Thermo Fisher	Cat# 10675112
Chloroform	Sigma Aldrich	Cat# 366927-2.5L
Methyl-tertiary butyl ether	Sigma Aldrich	Cat# 34875-2.5L
Ammonium acetate	Fluka	Cat# 17836-250G
Phosphatidylcholine C16:0-d31:C18:1	Avanti	
Glass coated 96 well plate		
SAG	Enzo Life Sciences	ALX-270-426-m001
LDN-193189	Cambridge Bioscience	2092-5
Recombinant Human FGF-8a Protein	R&D Systems	4745-F8-050
StemMACS Purmorphamine	Miltenyi Biotec	130-104-465
Bovine Serum Albumin 7.5%	Sigma-Aldrich	A8412
3',5'-Dibutyryl Cyclic AMP	Sigma-Aldrich	D0627-1G
L-ascorbic acid	Sigma-Aldrich	A4544-25G
DAPT	Tocris	2634/50
SB-431542	Tocris	1614/10
Y-27632	Tocris	1254/10
CHIR99021	Tocris	4423/10
2-Mercaptoethanol	Thermo Fisher Scientific	31350-010
Antibiotic-Antimycotic (100X)	Thermo Fisher Scientific	15240062
L-Glutamine 200mM	Thermo Fisher Scientific	25030081
N-2 Supplement (100X)	Thermo Fisher Scientific	17502048
B-27 Supplement (50X), minus Vitamin A	Thermo Fisher Scientific	12587-010
Geltrex LDEV-Free hESC-qualified	Life Technologies	A1413302
Knockout Replacement Serum	Life Technologies	10828-028
Neurobasal Media	Life Technologies	21103-049
StemPro Accutase	Life Technologies	A1110501
Dimethyl Sulfoxide	Merck	D2650-100ML
Recombinant Human BDNF	Peprotech	450-02
Recombinant Human GDNF	Peprotech	450-10
Recombinant Human TGF-b3	Peprotech	PHG9305
MEM Non-essential amino acids (100X)	Life Technologies	11140-035
Knockout DMEM	Life Technologies	10829-018
TeSR-E8	STEMCELL Technologies	05990
**Deposited Data**		
Raw and analysed data	This paper	
Analysis scripts used	This paper	
**Software**		
R	Open source	version 3.4.2

SIMCA	Umetrics	Version 13.0.4

Resource availability

Lead contact

Further information and requests for resources and reagents should be directed to the lead contact Albert Koulman (ak675@medschl.cam.ac.uk).

Material availability

This study did not generate any unique reagents.

Data and code availability

All of the code used in this study is shown in the supplemental information of the paper

Experimental model and subject details

Cell culture and dopaminergic differentiation

Human iPSCs were cultured in TeSR-E8 medium on Vitronectin coated plates. Cells were passaged with 0.5mM EDTA when reaching 70% confluency at a ratio of 1:6. Differentiation into dopaminergic neurons was performed according modify version of existing experimental protocols (Siddiqi et al., 2019, Kriks et al., 2011). In brief, iPSCs were first dissociated into single cells, plated at 150,000 cells/cm2 on Geltrex coated plates and grown for 11 days in Knockout Serum Replacement media (KSR) containing KO DMEM media, KSR (15%), Non Essential Amino Acids (1:100), 2-Mercaptoethanol (10 µM) and 2 mM L-glutamine. KSR medium was gradually changed to NNB medium containing Neurobasal medium, N2 (0.5X) and B27 (0.5X) and 2 mM L-glutamine from day 6. Media was changed to NB medium on day 12 containing Neurobasal medium, B27 (1X) and 2 mM L-glutamine. Medias were supplemented with LDN-193189 (100nM) from days 0-10; SB431542 (10 µM) from days 0-4; SAG (100 nM) from days 1-6; Purmorphamine (2 µM) from days 1-6; FGF8a (100 ng/ml) from days 1-6; and CHIR99021 (3 µM) from days 3-12. From Day 12 onwards, the following supplements were added: BDNF (20ng/ml), GDNF (20ng/ml), Ascorbic Acid (200 µM), TGFβ3 (1 ng/ml), dibutyryl cAMP (500 µM), and DAPT (10 µM). At day 21 cells were dissociated with StemPro Accutase and replated at 300,000 cells/cm2 in dishes pre-coated with Geltrex and fed every second day for 2 weeks before analysis.

Protocols for the differentiation of *in vitro* human dopamine neurons are known to generate heterogenous neuronal populations (Le Manno et al., 2016). To overcome this, we engineered human wild-type (WT) iPSC with a tyrosine hydroxylase (TH) red fluorescence protein (RFP) (Xia et al., 2017), with modifications. In order to express tagRFP-T under the influence of the endogenous TH promoter we introduced a P2A-tagRFP-T donor plasmid with homologous arms flanking the TH stop codon and an EF1A-Puromycin selection flanked by LoxP sites for drug selection. The targeting plasmid was introduced into iPS cells together with an sgRNA targeting the 3' end of the TH gene and Cas9 RNP by nucleofection to cause a double strand break near the stop codon. After Puromycin selection, transient expression of Cre was used to excise the Puromycin cassette. TH catalyses the hydroxylation

of L-DOPA (the rate limiting step in the synthesis of dopamine) and is considered a *bona fide* marker for dopamine neurons. After differentiation into dopamine neurons (Siddiqi et al., 2019; Kriks et al., 2011), fluorescence-activated cell sorting (FACS) was used to sort cells based on RFP expression. RFP positive dopamine neurons were individually sorted into multi-well plates, followed by LESA mass spectrometry.

TH-RFP engineering

iPSCs were engineered with a TH red fluorescence protein (RFP) reporter following previous methods (Xia et al., 2017) with modifications. We introduced a P2A-tagRFP-T donor plasmid with TH homologous arms and an EF1A-Puromycin selection flanked by LoxP sites for drug selection. The targeting plasmid was introduced into iPS cells together with a TH sgRNA (GACGCCGTGCACCTAGCCAA TGG) against the 3' end of the TH gene and Cas9 (HiFi) RNP by nucleofection (AMAXA 4D nucleofector, "CA 137" pulse code). Nucleofection was performed using the P3 Primary Cell 4D-Nucleofector X Kit L (cat. V4XP-3024) according to the manufacturer's instructions. Briefly, $1.5x10^6$cells were used for each nucleofection reaction together with 2μg of donor plasmid, 16μg of TH sgRNA and 20μg of HiFi Cas9. After Puromycin selection (0.25μg/ml for 10 days), transient expression of Cre (Tat-Cre, 4uM, 3 hours) was used to excise the Puromycin cassette. Clones were picked and genotyped for correct insertion using the following primers: GCCCTCGACCACCTTGATTCTCATGG and CCAAGACCAGACGTACCAGTCAGTC; GCCAGGGGCATCTTACAGAGTCTG and GGCCGACAAAGAGACCTACG; CAGGAGCTATGCCTCACGCATCCAG and GTGACGGTGATTGGGGCAGCAGAC.

Flow cytometry

Following differentiation until day 35, neuronal cultures were washed with PBS and incubated with StemPro Accutase (1X) for 20 min at 37ºC. An equal volume of Tripsin-EDTA (0.25%) was added followed by additional incubation at 37ºC for 10 min. Neurons were then gently dissociated into a single cell suspension with a P1000 pipette and reaction was neutralized by the addition of an equal volume of Neurobasal media containing Y-27632 (10µM). Cell suspension was then filtered through a 40µm FALCON Cell Strainer (Corning) to remove any undissociated cell clumps, and spun at 300xg for 5 minutes. Cells were resuspended in PBS and sorted based on their expression of RFP using a BD-Influx cell sorter (Becton-Dickinson, San Jose, CA, USA). Buffer used during sorting was BD FACSFlow (cat. 342003, BD Biosciences).

Method details

Liquid extraction surface analysis – mass spectrometry (LESA-MS)

Liquid extraction surface analysis (LESA) on TH positive dopamine neurons from the bottom of a well of a glass coated 96 well plate using a Triversa Nanomate (Advion, Ithaca, USA). Lipid extraction was achieved using a solvent of 20 mM ammonium acetate in isopropanol, methanol and chloroform (4:2:1, *v*:*v*:*v*), with 3 µl aspirated from the solvent reservoir with 1.0 µl subsequently dispensed onto the sample area and held there for 7 seconds before being re-aspirated and infused into the mass spectrometer for 90 seconds.

Data was acquire on an Exactive Orbitrap (Thermo, Hemel Hampstead, UK) with data acquired between 650-850 *m/z* in full scan in the positive ionisation mode. Parameters were optimised to maximise sensitivity (Supplemental Information 1) with the optimal settings being an infusion back pressure of 0.4 *psi*, 1.4 Kv electrospray current, mass accuracy of 100,000, a balanced AGC target, and an injection time of 100 m/s.

Quality control and extraction blanks

To help ensure that there was no bias in our experimental design an equal number of cells from each replicate was sorted onto each plate, with the samples from each replicate evenly distributed across the run order to help reduce within batch effects. Extraction blanks were created by diverting a droplet of FACS sheath fluid into 8 wells on each plate, with these wells subsequently treated identically to single cell samples. Quality control samples were generated by pooling additional neurons by FACS and spinning them down to produce a bulk pellet. This pellet was then extracted by adding 1.8 ml of MTBE:MeOH (5:1, v:v) and was then vortexed for 60 seconds after which 600 µl of water was added. Subsequently the sample was spun at 2000 rpm (845 × g) for 2 minutes to produce a phase separation with the upper organic phase removed. Prior to adding QC's to the plate, the material was analysed by LESA to assess concentration with MTBE added to reduce the concentration to give a signal within 2 orders of magnitude of the single cell signal. Once the QC sample was diluted 25 µl was added to 8 wells per plate and was subsequently dried under a continuous flow of nitrogen.

Optimisation of analytical method

Method optimisation was performed using a 1 nM solution of glycerophosphocholine (C16:0-d31/C18:1) dried down in a glass coated 96 well plate under a continuous stream of nitrogen. Liquid extraction surface analysis (LESA) was performed on these samples with a single parameter at a time with data collected from 5 replicates of each setting.

We demonstrated that at this low analyte concentration increasing infusion back pressure had no effect on signal abundance or signal to noise ratio (Supplemental Figure 6). The data also showed that increasing the electrospray current increased both signal abundance and signal to noise ratio up to a current of 1.4 kV, however when it was increased to 1.5kV we observe a slight increase in signal abundance but a slight reduction in signal to noise ratio (Supplemental Figure 7). When looking at the analysed mass window it can be seen that narrowing the mass range analysed increased both signal abundance and signal to noise ratio (Supplemental Figure 8). Reducing the solvent volume used to extract the sample from 7 µl to 3 µl increased both signal abundance and signal to noise ratio (Supplemental Figure 9). However, reducing this further to 1.5 µl actually reduced the measured signal abundance and signal to noise ratio (Supplemental Figure 9).

<u>Quantification and statistical analysis</u>

Data processing

Data processing was performed in R using an in-house pipeline (for code see Supplemental Information 2). Scans collected between 20 and 78 seconds were integrated and compared to

a list of 578 known lipid *m/z*'s (Supplemental Table 3) identifying the signal abundance and its deviation from the expected mass. Signal identification was done by comparing the abundance and mass deviation of the nearest signal to the target m/z in both FACS blanks and single cell samples. A signal was defined as present if it had a signal to noise ratio of > 1.5 and an average deviation of less than 10 ppm, if no signal was observed within 10 ppm the signal was defined as missing, with a positive signal identified in at least 5% of cells in two of the three analysed sample sets. To be defined as a measured signal a linear response between signal abundance and cell number (r>0.5) had to be observed (Table 1). Data processing for 560 samples and the accompanying 56 blanks and 56 QC samples took approximately 3 hours with signal identification and data clean up taking about 2 hours, which is comparable with other high throughput lipidomics methods (Harshfield et al., 2019).

Statistical Analysis

Prior to statistical analysis data was normalised to the total signal, this was done by calculating the abundance of all signals within each sample with all the measured signals then divided by the mean abundance. The mean abundance of each lipid was calculated for each biological replicate to give a 'population' lipid profile, which could be compared to single cell profiles. Multivariate analysis using principal component analysis (PCA) and partial least square discriminant analysis (PLS-DA) were performed with SIMCA v13.0.4 (Umetrics, Umeå, Sweden) all data was logarithmically transformed (base10) and scaled to unit variance (UV). The relationship between individual lipid species and given Y-variables were determined using generalised linear models (GLM) applied to the whole of the dataset, all models were calculated in 'R' (version 3.4.2).

Data and code availability

All R code used in this study is included in the supplemental information. All data will be upload to a publically available repository on publication of the manuscript.
